# Psychodynamics in Diabetes: The Relevance of Deepening the Symbolic in Treatment Adherence

**DOI:** 10.3389/fpsyg.2021.661211

**Published:** 2021-05-04

**Authors:** Francesco Marchini, Andrea Caputo, Alessio Convertino, Angela Napoli

**Affiliations:** ^1^Italian Centre of Analytical Psychology (CIPA), “Sapienza” University of Rome, Rome, Italy; ^2^Department of Dynamic and Clinical Psychology, and Health Studies, “Sapienza” University of Rome, Rome, Italy; ^3^Department of Experimental Medicine, “Sapienza” University of Rome, Rome, Italy; ^4^Department of Clinical and Molecular Medicine, “Sapienza” University of Rome, Rome, Italy

**Keywords:** diabetes, psychodynamics, alexithymia, defense mechanisms, transference–countertransference, psychoanalysis, grief

## Introduction

Among chronic diseases, diabetes is recognized as a major health risk negatively affecting the quality of life, with significant morbidity and mortality rates (World Health Organization, 2018), which is expected to rise to 552 million people by 2030, leading to considerable health expenditures (Whiting et al., 2011; Khan et al., 2020). Given its long-lasting nature and risky complications, diabetes requires continuous self-care including being active, eating healthily, taking medication, monitoring symptoms, solving problems, reducing risks, and healthy coping (Di Biase et al., 1997; Glasgow et al., 2008), thus making diabetes management and treatment adherence relevant challenges. In this regard, depressive and anxiety symptoms are often reported by patients with diabetes because of the disease-related distress including worry about complications, fear of hypoglycemia, and guilt feelings about uncontrolled blood glucose (Nouwen, 2015; Sartorius, 2018; Shinkov et al., 2018; Wardian et al., 2018). Overall, this difficulty complying with the stressful rigors of diabetes and required lifestyle changes tends to worsen glycemic control, resulting in higher hemoglobin A_1c_ levels and less optimal outcomes (Whithorth et al., 2016; Egan et al., 2017; Fukuda and Mizobe, 2017; Graham et al., 2020; Schmitt et al., 2021). Therefore, to promote higher patient self-care and treatment adherence, a primary task for healthcare professionals is reducing diabetes distress by improving patients' skills at regulating negative emotions (Coccaro et al., 2021). Emotion awareness and management in diabetes care are based on the capacity to deal with feelings and thoughts on both conscious and unconscious levels (Ivinson, 1995). From such a perspective, a psychodynamic approach can provide a significant contribution to a better understanding of the patient's emotional life. This article provides some basic psychodynamic insights with a practical value, focusing on the concepts of alexithymia, defense mechanisms, loss, mourning, and transference–countertransference dynamics in the therapeutic relationship. This may also help medical and nursing professions dealing with diabetes care to reconsider patient difficulties and communication through a new lens, thus improving clinical observation and care.

## Alexithymia

The psychosomatic tradition, especially influenced by the French School (Marty, 1963), has significantly contributed to the understanding of the influence of emotions and personality on health, focusing on impaired symbolic processes and poor imaginative capacity in subjects suffering from physical complaints. In the psychoanalytic literature, symbolic thinking refers to an unconscious mental process whereby sensory and somatic components of affective experience are translated into images and words, thus contributing to the development of a distinctive psychic self and the establishment of an intersubjective dialogue (Isaacs, 1948). Linking non-verbal experience with words allows the patient to differentiate between reality and fantasy and to acknowledge subjective feelings, so he can make sense of his experience (Bucci, 1997; Greenspan, 1997; Enckell, 2010). The conception of the body as a primary container of emotions (Bion, 1962; Anzieu, 1989), as well as the metaphor of “theater of the body” used by McDougall (1989), indicates that the body and its symptoms can represent unconscious conflicts that have not been mentalized. Thus, failures in symbolic thinking, the inability to translate emotions into words, may have a role in somatic suffering. Accordingly, the construct of alexithymia has been proposed to indicate the difficulty in describing and identifying emotions, accompanied by externally oriented thinking (Krystal, 1968; Nemiah and Sifneos, 1970; Taylor and Bagby, 2000). The role of alexithymia in the pathogenetic mechanisms of somatic illness has been widely documented in past research on several chronic conditions (Porcelli and Taylor, 2018; Martino et al., 2020c), including diabetes (Luminet et al., 2006; Martino et al., 2019). Recently, systematic empirical evidence has emerged about the presence of alexithymic traits in patients with diabetes and their association with psychological distress, in terms of depression, anxiety, and poor quality of life (Martino et al., 2020b). In addition, the limited resources of introspection in such patients may impair their ability to monitor body signals (Fantini-Hauwel, 2014), resulting in maladaptive coping strategies and reduced self-care (Topsever et al., 2006). This is confirmed by the robust correlation between alexithymia and poor glycemic control (Luca et al., 2015; Avci and Kelleci, 2016; Fares et al., 2019; Martino et al., 2020b). The difficulty in complying with the stressful rigors of diabetes management is also supported by the association between alexithymia and both unhealthy lifestyle behaviors (e.g., reduced physical activity) and increased hospitalization due to diabetes complications (Fares et al., 2019; Lai et al., 2019).

## Defense Mechanisms

Defense mechanisms play a relevant role in the emotional world of patients with diabetes. They represent unconscious strategies enacted to cope with the anxiety fueled by such a chronic condition, as well as factors that may perpetuate poor diabetic control over time (Ivinson, 1995; Hyphantis et al., 2005). Specifically, Ivinson (1995) discussed denial, displacement, and intellectualization as defenses patients use to keep diabetes-related painful feelings at bay and that impede the acceptance of a diagnosis of long-lasting illness. The relevance of such mechanisms in diabetes care has also been emphasized in light of their associations with depression and physical and mental well-being (Marchini et al., 2018, 2020; Martino et al., 2020a). However, their diverse nature should be considered because primitive defenses (e.g., projection) are associated with worse outcomes than more mature mechanisms (e.g., intellectualization, isolation, rationalization), which can be instead oriented to problem-solving and more advantageous to cope with illness (Hyphantis et al., 2005; La Grutta et al., 2013; Di Giuseppe et al., 2020; Martino et al., 2020a). In this regard, projection, aimed at justifying aggression against external sources of frustration, is associated with depressive feelings and reduced physical well-being, with negative implications for treatment adherence (Martino et al., 2020a) (e.g., the patient may attribute therapy inefficacy to the physician's incompetence rather than to one's poor compliance with recommendations). Instead, intellectualizing defenses, aimed at transforming conflicts into seemingly rational or intellectual explanations, correlate with both lower depression and greater health-related quality of life (Martino et al., 2020a) and may promote self-care and adherence (Marchini et al., 2020) (e.g., the patient may repeatedly justify high glucose levels by saying that he/she has had a stressful week compromising his/her diet regimen). Similar findings emerge about reversal. Minimizing the severity of perceived threats may reduce diabetes distress and contribute to proper glycemic control (Tripathy et al., 2012; Marchini et al., 2020; Martino et al., 2020a) (e.g., the patient may claim that he/she is unconcerned about having diabetes as it is not a serious disease). In this regard, compared to healthy controls, patients with diabetes resort to reversal to a greater extent, which appears to help them lessen depressive feelings (Marchini et al., 2020) but prevents effective diabetes management (Graham et al., 2020; Schmitt et al., 2021).

## Loss Mourning

From a psychodynamic perspective based on object relations theory and self-psychology (Greenberg and Mitchell, 1983), people who face chronic illness may experience feelings of loss and guilt related to a sense of self-defectiveness connected to a body symbolically damaged by a chronic condition (Goldstein, 2001; D'Alberton et al., 2012; Caputo, 2019, 2020a). Chronic disease, such as diabetes, may thus engender a process of “mourning” associated with negative feelings (e.g., anger, self-blame, self-criticism), which need to be processed to restore the damaged ego and consequently adapt to chronic illness. Indeed, patients with diabetes have to mourn the permanent loss of their formerly healthy body and may face death anxieties related to a life-threatening condition. In the context of diabetes care, Fonagy and colleagues highlighted the relevance of such psychological themes (e.g., damaged self-representation, deliberate self-punishment) connected with the sabotage of therapy and difficulties adhering to treatment (Moran and Fonagy, 1987; Moran et al., 1991; Fonagy and Moran, 1993). These clinical hypotheses find support in some recent pieces of research that demonstrate the association between self-blame and a poorer quality of life in persons with diabetes (Funuyet-Salas et al., 2020), as well as between increased levels of guilt and a tendency to cope through behavioral disengagement (Sarnie et al., 2020), with negative implications for health outcomes (Grylli et al., 2005; Yi-Frazier et al., 2015; Iturralde et al., 2017; Tanenbaum et al., 2020). In the psychodynamic realm, Marchini et al. (2018, 2020) have attempted to test an innovative model looking at “chronic illness as loss of a good self” within diabetes adaptation. The authors proposed four psychological “damaged ego strategies” (empathic identification, frustration, mania, and destructiveness) enacted to handle loss mourning in diabetes. Specifically, empathic identification involves acknowledging diabetes-related limitations but also preserving health maintenance; frustration relates to being trapped in a “betraying body” without hope for recovering a satisfying quality of life; mania implies denying diabetes-related implications and the relevance of self-care behaviors; and destructiveness concerns the enactment of angry feelings by sabotaging the received help. In this regard, only empathic identification, aimed at appreciating the good qualities of health, resulted positively associated with treatment adherence, whereas mania and destructiveness, indicating unprocessed mourning, negatively correlated with treatment adherence and quality of care, respectively (Marchini et al., in press).

## Transference–Countertransference Dynamics in the Therapeutic Relationship

Diabetes distress and painful related feelings such as guilt, shame, anger, self-blame, fear, and powerlessness (Abdoli et al., 2020), also connected to a condition of damaged ego, may be enacted within the relationship with healthcare providers, thus engendering transference–countertransference dynamics where elements personal to both client and therapist interact (Aron, 1996). Through projective identification (Ogden, 1979), an interpersonal process in which the recipient of an individual's projected feelings experiences pressure to think, feel, and behave in a manner congruent with such projections, professionals may become either overwhelmed or overidentified with the patient, thus leaving room for antitherapeutic behaviors (Frederickson, 1990; Evans, 2020). This may be also due to the inability to process the patient's death-related anxieties in the therapeutic relationship so that they can be reinternalized by the patient. Indeed, to better treat the patient, the professional must bear these anxieties, induced through projective identification; “digest” and metabolize them to understand the patient's suffering; and respond with empathy rather than revulsion. In this regard, some transference–countertransference dynamics can occur such as (1) wanting the doctor to offer omnipotent control over death; (2) equating the doctor with death, thus trying to control the “dangerous” doctor; (3) using denial and projection so that one's knowledge of death is projected into the doctor, who starts to feel crazy; or (4) manically identifying with death, taking pleasure in self-destruction so that the one who is terrified of death is the doctor. Observing these patterns of interaction can help the treatment professional to recognize the patient's defenses against the fear of dying and then respond from a position of deeper compassion (Frederickson, 2015). This appears in line with qualitative studies analyzing diabetologists' subjective experience with patients with diabetes, which have highlighted an overinvolvement in professional relationships accompanied by the fear of being overwhelmed (Sieradzki et al., 2006; Beverly et al., 2011; Craven et al., 2019). Such dynamics are accompanied by feelings of alertness including *preoccupation* described as separation anxiety and wish for contact, *control* based on hypervigilance and intrusiveness, and *fear* regarding an excessive closeness with an effort to distance, which emerged as countertransference responses to diabetes care challenges (Marchini et al., 2020). Moreover, non-adherence to medical treatment can lead diabetes care professionals to feel a sense of inefficacy, as if they lack the resources to address patients' needs and accomplish their professional goals (Sieradzki et al., 2006; Johansen et al., 2014; Craven et al., 2019). These results become more meaningful when put into the context of quantitative research pointing out that diabetes and prediabetes care are associated with worse mental health and higher levels of professional burnout (Bhutani et al., 2012; Seehusen et al., 2018; Gabbay and Barrett, 2020), suggesting the risks of dysfunctional therapeutic care relationships. In addition, the consequences of burnout syndromes can have a large impact on the quality of care and medical outcomes, as highlighted by a vast body of literature (Perlo et al., 2017; Patel et al., 2018).

## Discussion

The aim of this article was to provide physicians, nurses, and psychologists working in diabetic settings with some basic psychodynamic insights that could be addressed in education, training, and clinical supervision. Specifically, the promotion of reflective practices should be considered as an important tool to acknowledge the emotional life of patients with diabetes and healthcare professionals' reactions within care relationships (Caputo, 2020b). This can be useful to enhance patients' self-care and treatment adherence, as well as to prevent burnout syndromes in health providers (Abdoli et al., 2019). Also, the present article makes a significant contribution to the realm of psychological support and intervention of patients with such a chronic condition. As regards the relevance of alexithymic traits in persons with diabetes, recent studies have highlighted that mentalization-based interventions can increase diabetes outcomes and promote psychological well-being, especially in adolescents with type 1 diabetes (Iole Colombini and Schivalocchi, 2013; Thomakos et al., 2019; Costa-Cordella et al., 2020). To address alexithymia, the difficulty in identifying and describing emotions, the understanding of defense mechanisms preventing the patient from acknowledging his emotional world is of primary relevance. In this respect, collaborative care models involving the role of a psychologist within multidisciplinary diabetes care teams can be particularly useful (Johnson and Marrero, 2016) in conceptualizing patients' psychological modes of coping and delivering individualized care (Ivinson, 1995). Moreover, brief or supportive group-based psychodynamic interventions for promoting patients' adaptive coping styles have been demonstrated to lower anxiety, depressive symptoms, and diabetes-related problems (Simson et al., 2008; Carrillo-Alarcón et al., 2020). Given the challenges about the diabetes-related grief (Phillips, 2014; Carrillo-Alarcón et al., 2015; Fraser, 2020), supportive interventions can be proposed to improve stress management by allowing patients to recognize and accept their feelings of loss associated with their chronic condition. Indeed, patients are confronted with the actual loss of their formerly healthy body and physical functions as well as the eventual loss of their life, which need to be mourned. Along with this, they also have to mourn the symbolic losses imposed by diabetes in terms of self-image and lifestyle (Carrillo-Alarcón et al., 2015; Marchini et al., 2018) and to resolve the defensive process of disease-related personification (Shahar and Lerman, 2013). Moreover, when confronted with the patient's genuine helplessness and unconscious (or even conscious) wish that the doctor has omnipotent control over the condition, the doctor may become overinvolved (enacting the omnipotent transference) and thus burned out, or underinvolved and thus detached (resorting to defenses). Therefore, countertransference responses in the therapeutic relationship seem characterized by the coexistence of feelings of overinvolvement and alertness, suggesting the ongoing effort to restore patients' psychological integrity, as no definite cure is possible in such a chronic condition. From such a perspective, the doctor must also go through a mourning process, accepting chronic illness, helplessness, and death as facts, and accepting that potency as a doctor means mourning the loss of a self-image of the omnipotent doctor. Facing death-related anxieties thus requires a mutual mourning process involving both the doctor and the patient to avoid professional fatigue and maladaptive care relationships.

## Author Contributions

FM and ACa contributed to the article design and first writing. AN and ACo revised all the content. All the authors contributed to the final draft.

## Conflict of Interest

The authors declare that the research was conducted in the absence of any commercial or financial relationships that could be construed as a potential conflict of interest.
